# PET imaging of mGluR5 in Alzheimer’s disease

**DOI:** 10.1186/s13195-020-0582-0

**Published:** 2020-01-18

**Authors:** Adam P. Mecca, Julia W. McDonald, Hannah R. Michalak, Tyler A. Godek, Joanna E. Harris, Erika A. Pugh, Emily C. Kemp, Ming-Kai Chen, Arash Salardini, Nabeel B. Nabulsi, Keunpoong Lim, Yiyun Huang, Richard E. Carson, Stephen M. Strittmatter, Christopher H. van Dyck

**Affiliations:** 1 Alzheimer’s Disease Research Unit, Yale University School of Medicine, One Church Street, 8th Floor, New Haven, CT 06510 USA; 2 Department of Psychiatry, Yale University School of Medicine, New Haven, CT USA; 3 Department of Neuroscience, Yale University School of Medicine, New Haven, CT USA; 4 Department of Neurology, Yale University School of Medicine, New Haven, CT USA; 5 Department of Radiology and Biomedical Imaging, Yale University School of Medicine, New Haven, CT USA; 6 CNNR Program, Yale University School of Medicine, 295 Congress Avenue, Ste 431-435, New Haven, CT USA

**Keywords:** mGluR5, Glutamate receptor, Alzheimer’s disease, [^18^F]FPEB, PET

## Abstract

**Background:**

Metabotropic glutamate subtype 5 receptors (mGluR5) modulate synaptic transmission and may constitute an important therapeutic target in Alzheimer’s disease (AD) by mediating the synaptotoxic action of amyloid-β oligomers. We utilized the positron emission tomography (PET) radioligand [^18^F]FPEB to investigate mGluR5 binding in early AD.

**Methods:**

Sixteen individuals with amnestic mild cognitive impairment (MCI) due to AD or mild AD dementia who were positive for brain amyloid were compared to 15 cognitively normal (CN) participants who were negative for brain amyloid. Diagnostic groups were well balanced for age, sex, and education. Dynamic PET scans were acquired for 60 min, starting at 60 min after the initial administration of up to 185 MBq of [^18^F]FPEB using a bolus-plus-constant-infusion method (*K*_bol_ = 190 min). Equilibrium modeling with a cerebellum reference region was used to estimate [^18^F]FPEB binding (*BP*_ND_) to mGluR5. Analyses were performed with and without corrections for gray matter atrophy and partial volume effects.

**Results:**

Linear mixed model analysis demonstrated a significant effect of group (*p* = 0.011) and the group × region interaction (*p* = 0.0049) on *BP*_ND_. Post hoc comparisons revealed a significant reduction (43%) in mGluR5 binding in the hippocampus of AD (*BP*_ND_ = 0.76 ± 0.41) compared to CN (*BP*_ND_ = 1.34 ± 0.58, *p* = 0.003, unpaired *t* test) participants, and a nonsignificant trend for a reduction in a composite association cortical region in AD (*BP*_ND_ = 1.57 ± 0.25) compared to CN (*BP*_ND_ = 1.86 ± 0.63, *p* = 0.093) participants. Exploratory analyses suggested additional mGluR5 reductions in the entorhinal cortex and parahippocampal gyrus in the AD group. In the overall sample, hippocampal mGluR5 binding was associated with episodic memory scores and global function.

**Conclusions:**

[^18^F]FPEB-PET revealed reductions in hippocampal mGluR5 binding in early AD. Quantification of mGluR5 binding in AD may expand our understanding of AD pathogenesis and accelerate the development of novel biomarkers and treatments.

## Introduction

Metabotropic glutamate subtype 5 receptors (mGluR5) are seven-transmembrane G protein-coupled receptors located in excitatory synapses [1] and in glial cells [2]. They are distributed throughout the cortex and hippocampus where they modulate synaptic transmission [3, 4]. In rat brain, they are localized primarily postsynaptically [5, 6], but also presynaptically [7]. In primate prefrontal cortex, a substantial proportion are presynaptic [8]. In preclinical models of AD, mGluR5 has been hypothesized to mediate amyloid-β oligomer (Aβo) toxicity via several mechanisms, including promoting the clustering of Aβo as an extracellular scaffold for mGluR5 [9] and serving as a co-receptor for Aβo bound to cellular prion protein (PrP^c^) for postsynaptic activation of the tyrosine kinase Fyn [10, 11].

mGluR5 may also link Aβ pathology to tau pathology in AD [12]. Complexes of Aβo and PrP^c^ create a hydrogel phase that recruits mGluR5 [13], leading to activation of the tyrosine kinase Fyn [10]. This activation of Fyn leads to downstream tau phosphorylation [14]. Furthermore, functional tau is required for postsynaptic targeting of Fyn and subsequent excitotoxicity mediated by NMDA receptors [15]. The absence of functional tau prevents memory deficits and premature death in transgenic APP23 mice that develop Aβ plaques [15].

Recognition of mGluR5 as a mediator of AD pathology and a potentially important therapeutic target [16] has stimulated the investigation of mGluR5 expression and receptor binding in AD models. Two studies have measured mGluR5 changes in mouse models of AD using positron emission tomography (PET). Fang et al. investigated mGluR5 in AβPP transgenic mice (tg-ArcSwe) using [^11^C]ABP688-PET and reported no difference in binding compared to wild-type mice [17]. However, mGluR5 protein levels were increased in tg-ArcSwe mice when assessed with immunoblot. In a similar study, Lee et al. measured mGluR5 density in 5xFAD mice using [^18^F]FPEB-PET and immunoblot and observed lower mGluR5 binding and protein levels in the hippocampus and striatum compared to wild-type mice [18]. To our knowledge, no previous studies have investigated changes in mGluR5 receptor binding in living humans with AD.

In the present study, we utilized the PET radioligand [^18^F]FPEB to investigate mGluR5 binding in AD. To maximize statistical power in the setting of multiple regional comparisons, in our primary analyses, we focused on the hippocampus. This decision was based on postmortem [19, 20] and in vivo [21] evidence of early synaptic loss in this region in AD, as well as mGluR5 reductions in AD model mice [18]. We also examined a composite association cortical region, given the evidence for selective vulnerability of association cortex in AD [22, 23]. We hypothesized that mGluR5 binding in the hippocampus and association cortex would be reduced in AD compared to CN participants. Further exploratory analyses were conducted to determine whether mGluR5 binding was reduced in a wider range of regions. Finally, we examined the associations between mGluR5 binding in the hippocampus or association cortex with episodic memory performance and global function.

## Methods

### Study participants and design

Participants between 55 and 85 years old underwent a screening diagnostic evaluation to ensure eligibility. Individuals with AD dementia were required to meet diagnostic criteria for probable dementia due to AD according to the National Institute on Aging–Alzheimer’s Association [24], have a Clinical Dementia Rating (CDR) score of 0.5 to 1.0 points, and a Mini-Mental Status Examination (MMSE) score of 16 to 26 points, inclusive. Participants with MCI were required to meet research diagnostic criteria for amnestic MCI [25], have a CDR score of 0.5 points, and a MMSE score of 24 to 30 points, inclusive. Both participants with AD dementia and MCI were required to have impaired episodic memory as evidenced by a Logical Memory II (LMII) score of 1.5 standard deviations below an education-adjusted norm. Participants who were cognitively normal were required to have a CDR score of 0, a MMSE score greater than 26, and a normal education-adjusted LMII score. The Rey Auditory Verbal Learning Test (RAVLT) was also administered to generate an episodic memory score. All participants received a PET scan with [^11^C] Pittsburgh Compound B ([^11^C]PiB) to determine the presence of brain amyloid-β accumulation. The [^11^C]PiB PET scan was considered positive if both visual and quantitative criteria were met. Visual criteria entailed consensus of 2 experienced readers (APM and M-KC), and quantitative criteria required a [^11^C]PiB cerebral-to-cerebellar distribution volume ratio (*DVR*) of 1.40 or more in at least 1 AD-affected region of interest (ROI) [26]. The study protocol was approved by the Yale University Human Investigation Committee and Radiation Safety Committee. All participants provided written informed consent prior to participating in the study.

### Magnetic resonance imaging

Magnetic resonance imaging (MRI) was performed on a 3T Trio (Siemens Medical Systems, Erlangen, Germany) with a circularly polarized head coil. MRI acquisition consisted of a Sag 3D magnetization-prepared rapid gradient-echo (MPRAGE) sequence with 3.34-msec echo time, 2500-msec repetition time, 1100-msec inversion time, 7° flip angle, and 180 Hz/pixel bandwidth. Images are 256 × 256 × 176 with a pixel size of 0.98 × 0.98 × 1.0 mm. The MRI ensured that patients did not show evidence of infection, infarction, or other brain lesions. In addition, the MRI was used to define anatomy, to evaluate atrophy, and to perform partial volume correction (PVC).

### Positron emission tomography

#### Acquisition and reconstruction

PET scans were performed on the HRRT (207 slices, resolution < 3 mm full width half maximum), the highest resolution human PET scanner [27]. List-mode data were reconstructed using the MOLAR algorithm [28] with event-by-event motion correction based on an optical detector (Vicra, NDI Systems, Waterloo, Canada) [29].

Dynamic [^11^C]PiB scans were acquired for 90 min following administration of up to 555 MBq of tracer [30]. Dynamic [^18^F]FPEB scans were acquired for 60 min, starting at 60 min after the initial administration of up to 185 MBq of tracer using a bolus/infusion method (*K*_bol_ = 190 min) [31].

#### Image co-registration and MRI segmentation

Software motion correction was applied to the dynamic PET images using a mutual-information algorithm (FSL-FLIRT) to perform frame-by-frame registration to a summed image (60–70 min). A summed motion corrected PET image was registered to the participant’s MRI. The individual’s MRI was nonlinearly registered to a template MRI to obtain regions of interest (ROIs) defined in the automated anatomical labeling (AAL) template [32]. A full description of the ROIs can be found in Additional file 1. Transformations were performed with Bioimagesuite (version 2.5; www.bioimagesuite.com). MR images were segmented into gray matter (GM), white matter (WM), and cerebrospinal fluid (CSF) using FAST-FMRIB’s Automated Segmentation Tool (The Analysis Group, FMRIB, Oxford, UK). GM masking was performed by restricting ROIs using the GM segmentation mask.

#### Partial volume correction

PVC was performed using the Müller-Gärtner approach [33], according to previously described procedures [30]. Binary mask images of GM and WM were smoothed to the system resolution (~ 3 mm). For each dynamic PET frame, GM voxels were corrected for spill-in and spill-out of activity, assuming activity in CSF was zero and WM activity was uniform and was estimated from each image time frame.

#### Tracer kinetic modeling

For [^11^C]PiB image analysis, parametric images of binding potential (*BP*_ND_), the ratio at equilibrium of specifically bound radioligand to that of nondisplaceable radioligand in tissue [34], were generated using SRTM2 [35] with whole cerebellum as the reference region. *BP*_ND_ was calculated so that a value of 0 reflects no specific binding, i.e., tracer uptake no greater than that in the reference region. This is directly related to the *DVR* reported by other investigators [26], in that *DVR* = *BP*_ND_ + 1.

For [^18^F]FPEB image analysis, parametric images of *BP*_ND_ were generated using equilibrium methods [36] with PET data collected from 90 to 120 min postinjection and whole cerebellum reference region [31, 37]. Three sets of *BP*_ND_ values were extracted: (1) uncorrected *BP*_ND_ using the full AAL region, (2) uncorrected *BP*_ND_ from the AAL region masked only to include GM voxels, and (3) PVC *BP*_ND_, again with GM masking. We have previously evaluated a bolus plus constant infusion paradigm for equilibrium modeling of both the distribution volume (*V*_T_) and *BP*_ND_ for [^18^F]FPEB [31, 38] and demonstrated excellent test-retest reproducibility for both parameters [31]. Although a validated reference region is not available for mGluR5-specific radioligands [39], the estimation of *BP*_ND_ using a region with a small amount of specific binding may be useful with certain assumptions and limitations (see the “Discussion” section). One such assumption is that the specific binding in the reference region does not differ between diagnostic groups. In support of this assumption, we also compared *V*_T_ in whole cerebellum between our AD and CN groups. *V*_T_ was calculated as the tissue-to-plasma radioactivity ratio at equilibrium (90–120 min postinjection) and reflects total uptake (specific plus nonspecific binding).

### Whole brain PET and volumetric MRI analyses

Cortical reconstruction and volumetric segmentation were performed using Freesurfer (version 6.0, http://surfer.nmr.mhg.harvard.edu/) [40]. GM volume was normalized using estimated total intracranial volume [41]. For [^18^F]FPEB image analysis, Freesurfer was used to co-register the parametric *BP*_ND_ image to the MRI for each subject. [^18^F]FPEB *BP*_ND_ images were then sampled to the cortical surface and spatially smoothed using a 10 mm FWHM gaussian kernal.

### Statistical analyses

Statistical analyses were performed using SPSS version 21.0 (IBM Corp.) or Matlab R2015a Statistics Toolbox (Mathworks, Inc.). Primary analyses utilized linear mixed models to compare mGluR5 binding (*BP*_ND_) in the hippocampus and composite association cortex (within-participant factor) between AD and CN groups. The best-fitting variance-covariance structure, as determined by Bayesian information criterion, was compound symmetry. Secondary analyses utilized a similar model with exploratory regions listed in Table 2. Post hoc comparisons utilized unpaired *t* tests. To evaluate the contribution of GM tissue loss to mGluR5 reductions in AD, group differences in regional *BP*_ND_ after GM masking or PVC, as well as in regional GM volume, were also assessed using unpaired *t* tests. Additional exploratory analyses examined the relationships between hippocampal or association cortical *BP*_ND_ and episodic memory (average *z*-scores for LMII and RAVLT) and global function (CDR sum of boxes [CDR-SB]) in the combined sample with Pearson’s correlation. Tests were two-tailed and used *p* < 0.05 as a threshold for significance. Vertex-wise, whole cortical analyses were performed with general linear models using Freesurfer. Permutation was used to correct for multiple comparisons. The cluster-forming threshold was *p* < 0.01, and the cluster-wise threshold was *p* < 0.05.

## Results

### Participant characteristics

The study sample consisted of 31 participants—16 with amnestic MCI due to AD or mild AD dementia and 15 who were CN. Diagnostic groups were well balanced for age, sex, and education, and both groups were highly educated (Table 1). AD participants had clinical characteristics typical of amnestic MCI and mild AD dementia with MMSE = 24.6 ± 4.3 and CDR-global = 0.72 ± 0.26.

**Table 1:** Participant characteristics and test results

	Cognitively normal	Alzheimer’s disease
Participants (*n*)	15	16 (mild dementia, 8; MCI, 8)
Sex (M/F)	6/9	7/9
Age (years)	71.5 (8.4) (59–84)	73.1 (5.7) (63–82)
Education (years)	17.1 (2.3) (12–20)	16.7 (2.5) (12–20)
CDR-global	0 (0)	0.72 (0.26) (0.5–1)
CDR-SB	0 (0)	3.9 (2.2) (0.5–9.0)
MMSE	29.2 (1.2) (27–30)	24.6 (4.3) (17–29)
LMII	13.7 (3.8) (5–19)	1.9 (2.5) (0–7)
RAVLT-delay	11.7 (2.9) (4–15)	1.6 (2.5) (0–7)

Data are mean (SD) (range). *CDR-global* Clinical Dementia Rating global score, *CDR-SB* Clinical Dementia Rating sum of boxes, *MMSE* Mini-Mental State Examination, *LMII* Logical Memory II score, *RAVLT* Rey Auditory Verbal Learning Test

### mGluR5 binding in Alzheimer’s disease compared to cognitively normal participants

**Fig. 1:**
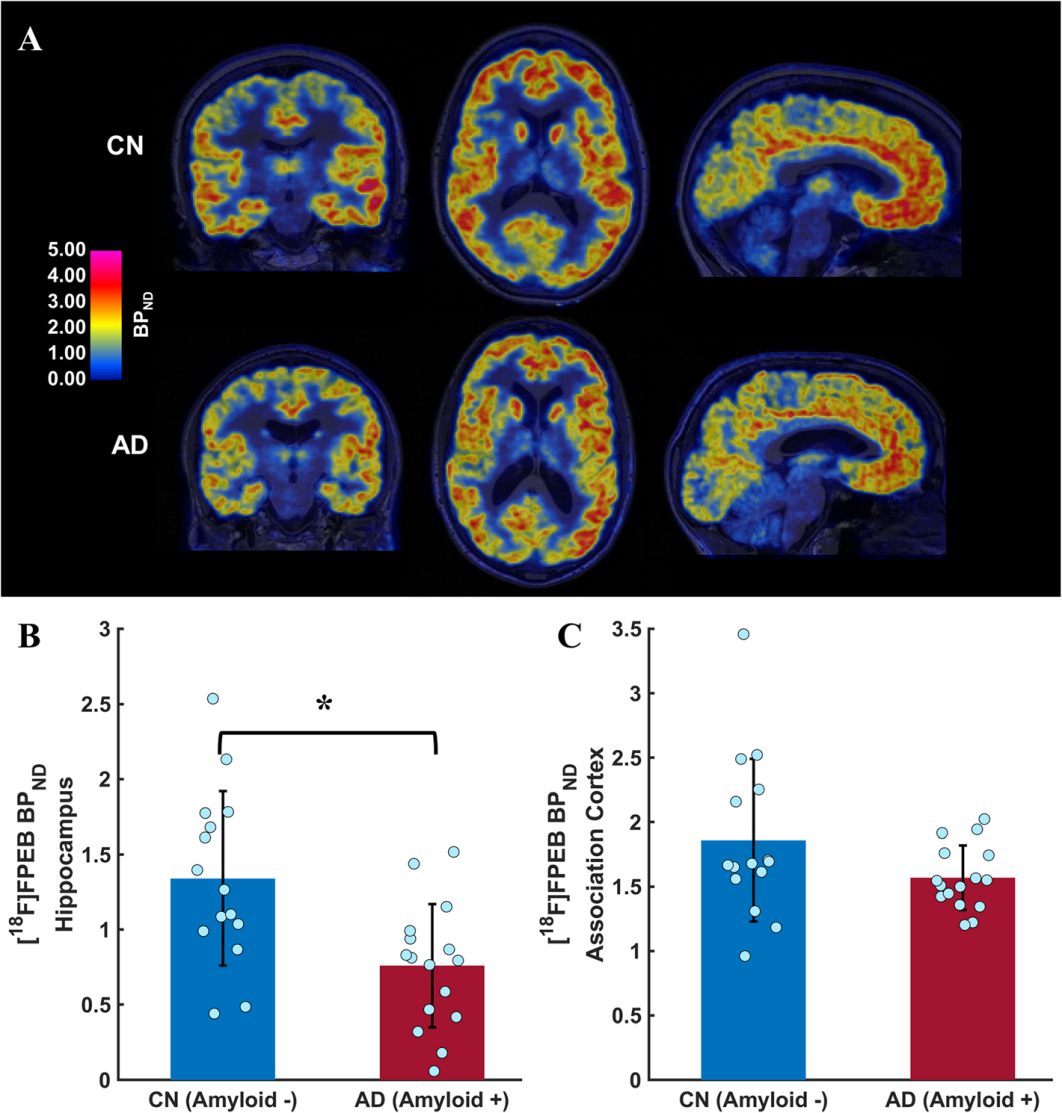
mGluR binding ([^18^F]FPEB *BP*_ND_) in AD and CN participants measured with [^18^F]FPEB-PET. **a** Coronial, axial, and sagittal images of [^18^F]FPEB parametric PET (*BP*_ND_) overlaid with T1 MRI scans in a representative CN (top row) and AD (bottom row) participant. The pseudocolor in PET images represents the intensity of [^18^F]FPEB binding (*BP*_ND_). Reductions of [^18^F]FPEB binding are most noticeable in the medial temporal lobe including hippocampus in the AD compared to the CN participants. However, mGluR5 binding appears to be lower in most cortical regions. Comparison of mGluR5 binding in the hippocampus (**b**) and association cortex (**c**) between AD and CN participants. AD participants—compared to CN participants—demonstrated significantly lower overall mGluR5 binding (*F* (1, 31) = 7.4, *p* = 0.011). In addition, there was a significant diagnostic group × region interaction (*F* (1, 31) = 9.2, *p* = 0.0049). Post hoc analyses revealed that hippocampal mGluR5 binding (*p* = 0.003, *t* test), but not association cortical mGluR5 binding (*p* = 0.093), was reduced in AD participants. Error bars represent standard deviations. *BP*_ND_, binding potential; CN, cognitively normal; AD, Alzheimer’s disease. *p < .05

All participants received one injection of [^18^F]FPEB (172 ± 21 MBq) with no significant difference in radioactivity (unpaired *t* test, *p* = 0.132) or mass dose (unpaired *t* test, *p* = 0.412) between groups. We observed no difference in whole cerebellar *V*_T_ from 90 to 120 min postinjection between AD (9.3 ± 1.8) and CN (8.6 ± 2.2) groups (unpaired *t* test, *p* = 0.309), supporting the use of cerebellum as the reference region in *BP*_ND_ calculations. Therefore, analyses were performed using parametric images normalized to whole cerebellum at equilibrium. Representative images of mGluR5 binding (*BP*_ND_) indicate receptor availability throughout the cortex and in subcortical structures (Fig. 1a). Linear mixed model analysis, including group (CN, AD), region (hippocampus, association cortex), and the group × region interaction as predictors, demonstrated a significant effect of group (*F* (1, 31) = 7.4, *p* = 0.011) and group × region (*F* (1, 31) = 9.2, *p =* 0.0049) on *BP*_ND_. Consistent with our hypothesis, we found a significant reduction (43%) in *BP*_ND_ in the hippocampus in AD (0.76 ± 0.41) compared to CN (1.34 ± 0.58) participants (*p* = 0.003, unpaired *t* test, Fig. 1b). However, we observed only a nonsignificant trend in mGluR5 binding in the association cortex between AD (1.57 ± 0.25) and CN (1.86 ± 0.63) participants (*p* = 0.093, unpaired *t* test, Fig. 1c).

A secondary linear mixed model analysis explored the effect of diagnostic group (AD, CN) on mGluR5 binding (*BP*_ND_) in a wider range of brain regions (Table 2). This analysis yielded a significant effect of group × region (*F* (11, 82) = 2.38, *p* = 0.013), but not group (*F* (1, 29) = 3.50, *p* = 0.072). Post hoc analyses showed significant reductions of mGluR5 (*BP*_ND_) in the entorhinal cortex (34% reduction, *p* = 0.002) and parahippocampal gyrus (26% reduction, *p* = 0.012) of AD participants (Table 2, unpaired *t* test, uncorrected for multiple comparisons).

**Table 2:** mGluR5 binding ([^18^F]FPEB *BP*_ND_) in exploratory brain regions of interest

Exploratory regions	Cognitively normal	Alzheimer’s disease	*p* value
	Mean *BP*_ND_ (SD)	Mean *BP*_ND_ (SD)	
Prefrontal cortex	1.89 (0.65)	1.64 (0.25)	0.155
Entorhinal cortex	1.62 (0.46)	1.07 (0.45)	0.002*
Parahippocampal gyrus	1.52 (0.48)	1.13 (0.32)	0.012*
Lateral temporal cortex	1.94 (0.64)	1.59 (0.27)	0.060
Lateral parietal cortex	1.80 (0.59)	1.50 (0.29)	0.077
Posterior cingulum	0.99 (0.61)	0.79 (0.25)	0.230
Precuneus	1.77 (0.57)	1.50 (0.26)	0.094
Occipital cortex	1.67 (0.53)	1.44 (0.21)	0.113
Caudate	1.26 (0.72)	1.07 (0.51)	0.391
Putamen	2.16 (0.77)	1.96 (0.30)	0.326
Thalamus	0.90 (0.50)	0.64 (0.25)	0.080

Data are mean (SD). *BP*_*ND*_ binding potential of [^18^F]FPEB in regions of interest. Cognitively normal (*n* = 15), Alzheimer’s disease (*n* = 16). *p* values are for post hoc two-tailed, unpaired *t* tests (uncorrected for multiplicity) performed after a linear mixed model analysis of *BP*_ND_ in multiple regions (within-subject factor) between CN and AD diagnostic groups

*p < .05

### Corrections for gray matter atrophy and partial volume effect

To evaluate the contribution of GM tissue loss to mGluR5 reductions in AD, we performed GM masking and PVC (Table 3). Hippocampal mGluR5 binding remained significantly lower in AD than in CN participants with GM masking (*BP*_ND_ = 1.30 ± 0.33 vs. 1.78 ± 0.61, *p* = 0.011) and PVC (*BP*_ND_ = 2.19 ± 0.45 vs. 2.69 ± 0.88, *p* = 0.0499). For the exploratory regional analyses, the reduction in mGluR5 remained significant after GM masking in the entorhinal cortex, but not in the parahippocampal gyrus. However, neither region retained significance after PVC (Table 3). This stepwise reduction in effect size with application of GM masking and PVC is consistent with both a dilution effect (i.e., as atrophy increases, GM volume within a region decreases) and a partial volume effect of GM atrophy on mGluR5 binding. To further elucidate these effects, we also performed a volumetric MRI analysis to assess GM volume differences between groups. This analysis demonstrated significant reductions in GM volume in the AD participants that were largest in the hippocampus and entorhinal cortex, but also present in the composite association cortex, parahippocampal gyrus, lateral temporal cortex, posterior cingulum, and occipital cortex (Additional file 1: Table S1).

**Table 3:** mGluR5 binding ([^18^F]FPEB *BP*_ND_) in brain regions of interest

	*BP*_ND_—gray matter masked			*BP*_ND_—partial volume corrected		
	CN, mean (SD)	AD, mean (SD)	*p*	CN, mean (SD)	AD, mean (SD)	*p*
Primary region						
Hippocampus	1.78 (0.61)	1.30 (0.33)	0.011*	2.69 (0.88)	2.19 (0.45)	0.0499*
Composite association cortex	2.38 (0.76)	2.04 (0.29)	0.111	4.39 (1.30)	3.99 (0.47)	0.258
Exploratory regions						
Prefrontal cortex	2.43 (0.78)	2.15 (0.29)	0.197	4.48 (1.34)	4.10 (0.49)	0.310
Entorhinal cortex	1.99 (0.72)	1.52 (0.43)	0.034*	3.18 (1.18)	2.69 (0.54)	0.144
Parahippocampal gyrus	1.96 (0.69)	1.58 (0.33)	0.052	3.26 (1.13)	2.86 (0.44)	0.199
Lateral temporal cortex	2.43 (0.81)	2.03 (0.31)	0.075	4.24 (1.34)	3.78 (0.45)	0.204
Lateral parietal cortex	2.32 (0.72)	1.97 (0.31)	0.091	4.44 (1.26)	4.01 (0.52)	0.211
Posterior cingulum	1.65 (0.64)	1.42 (0.30)	0.219	3.23 (1.05)	3.01 (0.45)	0.448
Precuneus	2.15 (0.66)	1.82 (0.27)	0.079	4.08 (1.16)	3.71 (0.47)	0.249
Occipital cortex	2.02 (0.59)	1.77 (0.25)	0.120	3.82 (1.03)	3.57 (0.41)	0.388
Caudate	2.17 (0.86)	1.98 (0.42)	0.439	3.41 (1.26)	3.14 (0.46)	0.427
Putamen	2.79 (0.91)	2.57 (0.35)	0.381	4.44 (1.37)	4.19 (0.64)	0.514
Pallidum	0.96 (0.41)	0.81 (0.42)	0.312	2.50 (1.18)	2.30 (0.83)	0.599
Thalamus	1.38 (0.61)	1.12 (0.27)	0.118	2.40 (0.91)	2.04 (0.41)	0.162

Data are mean (SD). *BP*_*ND*_ binding potential of [^18^F]FPEB in regions of interest, *CN* cognitively normal (*n* = 15), *AD* Alzheimer’s disease (*n* = 16). *p* values are for post hoc two-tailed, unpaired *t* tests (uncorrected for multiplicity) performed after a linear mixed model analysis of *BP*_ND_ in multiple regions (within-subject factor) between CN and AD diagnostic groups

### Association between mGluR5 binding and episodic memory performance and global function

**Fig. 2:**
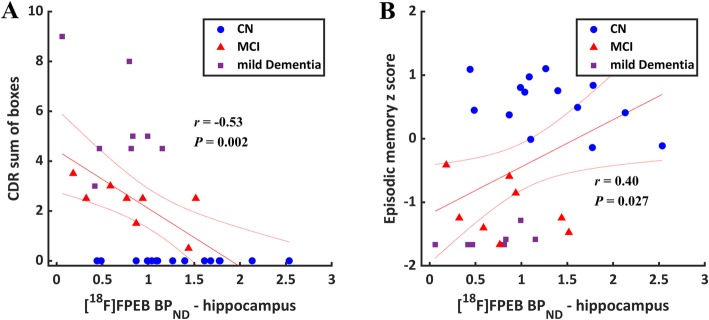
Association of mGluR5 binding ([^18^F]FPEB *BP*_ND_) in the hippocampus with Clinical Dementia Rating (CDR) sum of boxes and episodic memory. Reduced hippocampal mGluR5 binding was associated with more severe disease (*r* = − 0.53, *p* = 0.002) measured by CDR sum of boxes (**a**) and lower composite episodic memory scores (**b**) (*r* = 0.40, *p* = 0.027) in the overall sample. Episodic memory performance is the average of *z*-scores for CVLT free delayed recall and Logical Memory II. The figure displays linear regression line with its 95% confidence interval. CDR, Clinical Dementia Rating

Pearson’s correlations were performed to assess the relationship between mGluR5 binding and clinical assessments. Statistically significant correlations were found between hippocampal *BP*_ND_ and CDR-SB (*r* = − 0.53, *p =* 0.002) and episodic memory performance (*r* = 0.40, *p =* 0.027; Fig. 2). No significant correlations were observed between association cortical *BP*_ND_ and CDR-SB (*r* = − 0.27, *p =* 0.143) or episodic memory performance (*r* = 0.14, *p =* 0.451).

### Whole brain analyses of mGluR5 binding

**Fig. 3:**
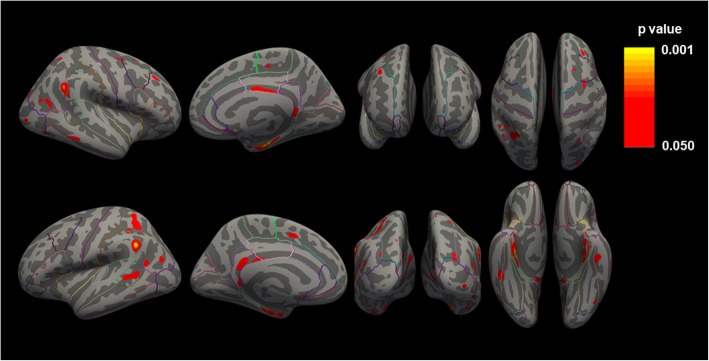
Whole cortex comparison of mGluR5 binding ([^18^F]FPEB *BP*_ND_) between AD and CN groups. *p* values are for vertex-wise comparisons between AD and CN groups uncorrected for multiple comparisons and thresholded at *p* < .05. Significant vertices are represented in pseudocolor. All displayed clusters are for the contrast CN > AD. *BP*_ND_, binding potential; CN, cognitively normal; AD, Alzheimer’s disease

Further exploratory analyses were performed to compare mGluR5 binding in AD and CN participants for both the whole cortex (surface-based approach) and all FreeSurfer Desikan-Killiany regions. For the surface-based analysis, there were no significant differences between AD and CN groups when a cluster-wise correction for multiple comparisons was applied. In an uncorrected surface-based analysis, the cortical pattern of mGluR5 binding in AD included significant reductions in the entorhinal cortex and posterior cingulum. There were also clusters of reduced signal throughout the cortices more broadly (Fig. 3, Additional file 1: Table S2).

For the analysis of all FreeSurfer regions, the effect size (Cohen’s *d*) to detect a difference in *BP*_ND_ between AD and CN groups was calculated (Additional file 1: Figure S1). Consistent with the primary regional analyses, the largest effect sizes were found in the medial temporal lobe. Additional file 1: Table S3 presents group differences (unpaired *t* tests) for all ROIs included in Additional file 1: Figure S1.

## Discussion

We used PET to investigate [^18^F]FPEB binding (*BP*_ND_) in early AD and observed a significant 43% reduction of mGluR5 availability in hippocampus but only a nonsignificant trend in a composite association cortical region. Exploratory analyses in a wider range of ROIs also suggested lower mGluR5 binding in the entorhinal cortex and parahippocampal gyrus. Reduction in mGluR5 availability in the hippocampus, but not entorhinal cortex or parahippocampal gyrus, remained significant after corrections for GM atrophy and partial volume effects. Additional exploratory analyses suggested that hippocampal mGluR5 binding was associated with episodic memory performance and inversely associated with global function (CDR-SB) in the overall sample.

### Comparison with AD model mouse and postmortem human studies

This is the first investigation of mGluR5 availability in living AD subjects. Previous studies of mGluR5 expression have been limited to mouse models of AD and a single small postmortem report. Fang et al. investigated changes in mGluR5 expression in AβPP transgenic mice (tg-ArcSwe) with ex vivo immunoblotting and in vivo [^11^C]ABP688-PET imaging. Immunoblot assays showed that brain mGluR5 levels tended to be upregulated in tg-ArcSwe mice compared with wild-type mice, although these changes were not discernible with PET [17]. By contrast, Lee et al. measured mGluR5 density in 5xFAD mice using [^18^F]FPEB-PET and immunoblot and observed lower mGluR5 binding and protein levels in the hippocampus and striatum compared to wild-type mice [18]. The reasons for these divergent findings in mouse models of AD are unclear. However, 5xFAD mice recapitulate more features of AD, including loss of neurons and a reduction of several synaptic markers [42], which may explain the greater similarity to our results with [^18^F]FPEB-PET in human AD. The only postmortem study of mGluR5 binding in AD by Müller Herde et al. [43] utilized [^18^F]PSS232 autoradiography and reported increases in the frontal cortex (5.2-fold) and hippocampus (2.5-fold) in 6 patients with severe AD compared to 6 controls. The authors speculate that neuroinflammation may lead to mGluR5 upregulation in severe AD and note that these results may not apply to early-stage AD, which may indeed explain the discrepancy with our results using [^18^F]FPEB-PET. However, further postmortem and in vivo research at different stages of AD will be necessary to elucidate these issues.

### Relevance for AD pathogenesis

The significance of reduced hippocampal [^18^F]FPEB binding in early AD is unclear. Hippocampal reductions in mGluR5 may simply be the product of nonspecific synaptic loss, which would explain the similarity of these results (43% reduction in hippocampal *BP*_ND_) with our recent findings with the synaptic PET tracer [^11^C]UCB-J in a comparable early AD sample [21]. That study demonstrated a 41% reduction in hippocampal *BP*_ND_, consistent with postmortem reports of hippocampal synaptic loss in MCI and mild AD [19, 20]. Alternatively, the presence of mGluR5 may influence the regional pattern of synaptic loss, given the evidence for involvement of this receptor in AD pathogenesis. mGluR5 has been hypothesized to mediate Aβo synaptotoxicity by a number of mechanisms, including promoting the clustering of Aβo as an extracellular scaffold for mGluR5 [9] and serving as a co-receptor for Aβo bound to PrP^c^ for postsynaptic activation of the tyrosine kinase Fyn [11, 44]. If Aβo synaptotoxicity occurs preferentially at mGluR5 sites, then this might also account for the synaptic pattern of mGluR5 reductions in the present study. Multitracer PET imaging studies with [^18^F]FPEB and [^11^C]UCB-J may be able to dissociate the regional pattern of mGluR5 and synaptic losses in early AD.

### Corrections for brain atrophy

We have presented mGluR5 binding results for [^18^F]FPEB-PET both with and without correction for AD-related decreases in regional brain volumes. We calculated the *BP*_ND_ for AAL-derived ROIs and repeated this calculation using a GM mask [30]. Finally, we performed PVC with the Müller-Gärtner algorithm to correct for GM signal loss (spill-out) due to atrophy [30, 33]. PVC typically has its greatest impact in those ROIs with large differences in GM volume between AD and CN groups (Additional file 1: Table S1) where spill-out could falsely lower *BP*_ND_. As expected, values of *BP*_ND_ increased with application of these correction methods (Table 2 and Table 3), but group differences in hippocampal mGluR5 binding remained significant—albeit with decreased magnitude. Our results suggest that lower hippocampal mGluR5 binding in AD is driven partly by a loss of GM volume but that a decrease in receptor density is also present in the remaining tissue. Among the outcome measures presented, the optimal one may depend on the particular purpose. Uncorrected analyses have greater sensitivity when mGluR5 imaging is utilized as a biomarker of disease presence or progression and may introduce less measurement error. This measure also summarizes the net loss of mGluR5, i.e., a combination of tissue loss and loss of mGluR5 in the remaining tissue. Corrections for GM loss and partial volume effects are better suited to determine group differences in receptor concentrations and may permit comparison to in vitro studies using animal models and postmortem human brain tissue.

### Assumptions and limitations of mGluR5 receptor quantification with *BP*_ND_

In this study, we quantified [^18^F]FPEB binding to mGluR5 using *BP*_ND_ generated from equilibrium modeling [36] with whole cerebellum as the reference region. We have previously evaluated a bolus plus constant infusion paradigm for equilibrium modeling of *V*_T_ and *BP*_ND_ for [^18^F]FPEB [31, 37, 38] and demonstrated excellent test-retest reproducibility for both parameters [31]. A major strength of *BP*_ND_ over *V*_T_—particularly for an older, AD population—is that it does not require arterial or venous blood sampling and is less susceptible to errors in the input function [28, 40]. The major limitation of *BP*_ND_ for mGluR5 quantification with [^18^F]FPEB is that it assumes a validated reference region with negligible specific binding. Although cerebellum is the region with the least mGluR5 specific binding, a small but measurable mGluR5 signal is observed in human cerebellum [33]. This will cause *BP*_ND_ values to be underestimated and the magnitude of percent group differences to be overestimated. Nonetheless, the estimation of *BP*_ND_ using a region with a small amount of specific binding may be useful with certain assumptions—in particular, that specific binding in the reference region does not differ between diagnostic groups. Importantly, we observed no significant difference in cerebellar *V*_T_ (reflecting specific plus nonspecific binding) between AD and CN groups.

## Conclusion

We observed reduced hippocampal mGluR5 binding with [^18^F]FPEB-PET in early AD compared to CN participants. Exploratory analyses suggested that these reductions may extend to other medial temporal lobe structures. Further study is needed to define the regional pattern and temporal course of mGluR5 alterations in AD, as well as the associations with cognitive and functional status. Quantification of [^18^F]FPEB binding to mGluR5 in AD may expand our understanding of AD pathogenesis and aid in the development of novel biomarkers and treatments.

## Supplementary information

**Additional file 1: **Supplemental Methods. Description of AAL regions use to construct composite ROIs. **Table S1.** Gray Matter Volume (cm^3^) in brain regions of interest. Gray matter volume comparison in AD and CN groups. **Table S2A.** Left hemisphere surface-based analysis of mGluR5 binding. List of freesurfer ROI group differences in the left hemisphere. **Table S2B.** Right hemisphere surface-based analysis of mGluR5 binding. List of freesurfer ROI group differences in the right hemisphere. **Figure S1.** Effect size maps of [^18^F]FPEB binding (*BP*_ND_) to mGluR5 in AD compared to CN participants. **Table S3.** mGluR5 binding in all FreeSurfer regions.

## Data Availability

The datasets used and/or analyzed during the current study are not publicly available due to ongoing analysis and manuscript preparation but are available from the corresponding author on reasonable request.

## Abbreviations

AAL: Automated anatomical labeling
AD: Alzheimer’s disease
Aβo: Amyloid-β oligomer
tg-ArcSwe: AβPP transgenic mice
*BP*_ND_: Binding potential
PiB: Pittsburgh Compound B
CDR: Clinical Dementia Rating
CN: Cognitively normal
CSF: Cerebrospinal fluid
DVR: Distribution volume ratio
GM: Gray matter
LMII: Logical Memory II
MCI: Mild cognitive impairment
mGluR5: Metabotropic glutamate subtype 5 receptor
MMSE: Mini-Mental Status Examination
MPRAGE: Magnetization-prepared rapid gradient-echo
MRI: Magnetic resonance imaging
PET: Positron emission tomography
PrP^c^: Cellular prion protein
PVC: Partial volume correction
RAVLT: Rey Auditory Verbal Learning Test
ROI: Region of interest
*V*_T_: Distribution volume
WM: White matter
