# Supplementary material for: PET imaging of mGluR5 in Alzheimer’s disease
Source: Alzheimers Res Ther. 2020 Jan 18;12:15. doi: 10.1186/s13195-020-0582-0 (PMC6969979; doi:10.1186/s13195-020-0582-0)
Supplement: Supplementary file 1 — Additional file 1: Supplemental Methods. Description of AAL regions use to construct composite ROIs. Table S1. Gray Matter Volume (cm3) in brain regions of interest. Gray matter volume comparison in AD and CN groups. Table S2A. Left hemisphere surface-based analysis of mGluR5 binding. List of freesurfer ROI group differences in the left hemisphere. Table S2B. Right hemisphere surface-based analysis of mGluR5 binding. List of freesurfer ROI group differences in the right hemisphere. Figure S1. Effect size maps of [18F]FPEB binding (BPND) to mGluR5 in AD compared to CN participants. Table S3. mGluR5 binding in all FreeSurfer regions. [file 13195_2020_582_MOESM1_ESM.docx]

**Supplemental Methods**

**Regions of interest.** [^18^]F-FPEB PET regions of interest (ROIs) were defined using the automated anatomical labeling (AAL) algorithm for SPM2 (*1*). For the primary analyses, using the published list of AAL regions, a hippocampal ROI was defined to include bilateral regions 42 – 43. The composite association cortical region comprised bilateral frontal regions 3 – 16, parietal regions 59 – 62, temporal regions 81 – 82, 85 – 86, 89 – 90, and occipital regions 49 – 54. The cerebellar reference ROI included bilateral regions 91 – 115.

For the exploratory analyses, the prefrontal ROI included bilateral AAL regions 3 – 34, the parahippocampal ROI regions 39 – 40, the lateral temporal ROI regions 79 – 90, the lateral parietal ROI regions 59 – 66, the posterior cingulate ROI regions 35 – 36, the precuneus ROI regions 67 – 68, the occipital ROI regions 43 – 56, the caudate ROI regions 71 – 72, the putamen ROI regions 73 – 74, and the thalamus ROI regions 59 – 60. The entorhinal cortical region was defined based on the Desikan-Killiany atlas(*2*).

**References.**

**1.** Tzourio-Mazoyer N, Landeau B, Papathanassiou D, et al. Automated anatomical labeling of activations in SPM using a macroscopic anatomical parcellation of the MNI MRI single-subject brain. *Neuroimage.* 2002;15:273-289.

**2.** Desikan RS, Segonne F, Fischl B, et al. An automated labeling system for subdividing the human cerebral cortex on MRI scans into gyral based regions of interest. *Neuroimage.* 2006;31:968-980.

| **Supplemental Table 1. Gray Matter Volume (cm^3^) in brain regions of interest** | | | |
| --- | --- | --- | --- |
|  | **Cognitively Normal** | **Alzheimer’s disease** |  |
| **Region** | **Mean Volume (SD)** | **Mean Volume (SD)** | ***P*** |
| **Hippocampus** | 7.5 (0.9) | 6.1 (0.8) | <0.001* |
| **Association cortex** | 208.2 (16.8) | 196.4 (14.7) | 0.046* |
| **Prefrontal cortex** | 92.6 (7.7) | 91.1 (6.1) | 0.569 |
| **Entorhinal cortex** | 3.0 (0.6) | 2.3 (0.4) | 0.002* |
| **Parahippocampal gyrus** | 3.5 (0.5) | 3.0 (0.5) | 0.015* |
| **Lateral temporal cortex** | 54.7 (6.5) | 48.2 (5.5) | 0.006* |
| **Lateral parietal cortex** | 40.5 (3.2) | 38.6 (3.8) | 0.136 |
| **Posterior cingulum** | 9.5 (0.8) | 8.7 (0.8) | 0.012* |
| **Precuneus** | 16.5 (1.2) | 15.6 (1.6) | 0.093 |
| **Occipital cortex** | 20.4 (1.9) | 18.5 (2.0) | 0.010* |
| **Caudate** | 6.3 (0.5) | 6.5 (0.8) | 0.472 |
| **Putamen** | 8.6 (1.5) | 8.2 (1.1) | 0.354 |
| **Thalamus** | 12.8 (1.2) | 12.1 (1.3) | 0.165 |

Data are mean (SD). Cognitively normal (n = 15), Alzheimer’s disease (n = 16). *P* values are for two-tailed, unpaired t-tests (uncorrected for multiplicity). **P* < 0.05

| **Supplemental Table 2A. Left hemisphere surface-based analysis of mGluR5 binding** | | | | | | | |
| --- | --- | --- | --- | --- | --- | --- | --- |
| **Cluster** | **ROI** | ***P* Max** | **Area (mm2)** | **MNI X** | **MNI Y** | **MNI Z** | **CW *P*** |
| 1 | Parahippocampal | 0.002 | 1644.22 | -20.8 | -17.9 | -27.4 | 0.136 |
| 2 | Superior parietal | 0.006 | 1068.45 | -28.7 | -48.9 | 42.6 | 0.165 |
| 3 | Fusiform | 0.019 | 584.38 | -41.1 | -64.7 | -13.1 | 0.220 |
| 4 | Isthmus cingulate | 0.006 | 580.63 | -8.7 | -43.5 | 24.5 | 0.222 |
| 5 | Bankssts | 0.006 | 522.69 | -50.3 | -49.1 | 3.8 | 0.230 |
| 6 | Supramarginal | 0.001 | 304.42 | -55.1 | -50.9 | 29.2 | 0.305 |
| 7 | Inferior parietal | 0.021 | 293.04 | -29.7 | -77.1 | 14.9 | 0.310 |
| 8 | Superior parietal | 0.021 | 221.63 | -19.3 | -80.9 | 41 | 0.356 |
| 9 | Superior frontal | 0.011 | 198.64 | -9.6 | 4.4 | 46.5 | 0.370 |
| 10 | Supramarginal | 0.015 | 173.13 | -44.3 | -54.5 | 38.8 | 0.385 |
| 11 | Precentral | 0.023 | 164.81 | -27.3 | -13.9 | 59.2 | 0.393 |
| 12 | Inferior parietal | 0.008 | 151.92 | -41 | -56 | 12.3 | 0.407 |
| 13 | Inferior parietal | 0.012 | 148.23 | -40.8 | -69.9 | 19.8 | 0.413 |
| 14 | Superior temporal | 0.028 | 147.14 | -44.3 | -2.2 | -21.1 | 0.413 |
| 15 | Precentral | 0.035 | 124.21 | -42 | 0.4 | 40.1 | 0.441 |
| 16 | Middle temporal | 0.030 | 121.54 | -54.6 | -18.2 | -17.1 | 0.447 |
| 17 | Middle temporal | 0.032 | 79.28 | -47.6 | -31.1 | -8.4 | 0.525 |
| 18 | Superior parietal | 0.037 | 79.08 | -10.2 | -66.8 | 58.5 | 0.527 |
| 19 | Posterior cingulate | 0.038 | 73 | -16.3 | -36.3 | 38.9 | 0.539 |
| 20 | Superior frontal | 0.037 | 67.52 | -20.3 | 28 | 52 | 0.547 |
| 21 | Caudal middle frontal | 0.030 | 58.42 | -34.1 | 22.2 | 47.2 | 0.574 |
| 22 | Rostral middle frontal | 0.038 | 57.78 | -30.8 | 31.9 | 37.4 | 0.576 |
| 23 | Superior parietal | 0.036 | 38.66 | -12 | -53.8 | 63 | 0.634 |
| 24 | Superior temporal | 0.039 | 34.88 | -49.2 | -12.2 | -6.5 | 0.647 |
| 25 | Postcentral | 0.034 | 28.95 | -39.9 | -22 | 46 | 0.666 |
| 26 | Superior frontal | 0.049 | 1.82 | -13 | 20.8 | 34.3 | 0.765 |
|  |  |  |  |  |  |  |  |

All cluster contrasts were Cognitively Normal > Alzheimer's disease. ROI = Region of Interest, Area = area of the cluster in mm2, [MNI_X MNI_Y MNI_Z] = MNI305 coordinates of the cluster, *P* Max = maximum *P* value within the cluster prior to correction for multiple comparisons, CW *P* = cluster-wise *P* value corrected for multiple comparisons using permutation, Bankssts = banks of the superior temporal sulcus

| **Supplemental Table 2B. Right hemisphere surface-based analysis of mGluR5 binding** | | | | | | | |
| --- | --- | --- | --- | --- | --- | --- | --- |
| **Cluster** | **ROI** | ***P* Max** | **Area (mm2)** | **MNI X** | **MNI Y** | **MNI Z** | **CW *P*** |
| 1 | Fusiform | 0.013 | 527.86 | 43.8 | -39.3 | -18.2 | 0.235 |
| 2 | Entorhinal | 0.001 | 486.27 | 21.3 | -13.9 | -27.7 | 0.244 |
| 3 | Superior parietal | 0.018 | 469.46 | 26.7 | -78.8 | 14.9 | 0.25 |
| 4 | Inferior parietal | 0.001 | 456.24 | 53 | -50.4 | 27.6 | 0.253 |
| 5 | Inferior parietal | 0.015 | 407.58 | 40.5 | -57.6 | 15 | 0.263 |
| 6 | Posterior cingulate | 0.011 | 367.2 | 5.3 | -7.7 | 30.3 | 0.278 |
| 7 | Middle temporal | 0.010 | 245.86 | 57.6 | -40.1 | -14.1 | 0.352 |
| 8 | Lateral occipital | 0.014 | 204.8 | 45.8 | -64.7 | -9.7 | 0.38 |
| 9 | Isthmus cingulate | 0.014 | 200.46 | 8.5 | -44.1 | 8.7 | 0.385 |
| 10 | Precentral | 0.016 | 189.52 | 29.4 | -8.4 | 45.4 | 0.397 |
| 11 | Rostral middle frontal | 0.011 | 189.3 | 31.3 | 29.6 | 38.8 | 0.397 |
| 12 | Superior frontal | 0.024 | 172.59 | 21.4 | 10.9 | 49.8 | 0.412 |
| 13 | Superior parietal | 0.019 | 157.2 | 27.7 | -54.5 | 58.7 | 0.424 |
| 14 | Lateral occipital | 0.011 | 124.6 | 37.4 | -88.2 | -2.7 | 0.464 |
| 15 | Precentral | 0.031 | 97.75 | 25.1 | -22.3 | 53.6 | 0.509 |
| 16 | Superior parietal | 0.023 | 95.87 | 11.4 | -59.9 | 61.8 | 0.512 |
| 17 | Paracentral | 0.019 | 91.55 | 12.8 | -21.2 | 47.4 | 0.518 |
| 18 | Fusiform | 0.028 | 71.98 | 28.4 | -42.9 | -18.3 | 0.563 |
| 19 | Bankssts | 0.035 | 65.87 | 49.8 | -37.7 | -5.2 | 0.581 |
| 20 | Superior parietal | 0.023 | 52.07 | 29.4 | -50.5 | 40.1 | 0.613 |
| 21 | Lateral orbitofrontal | 0.033 | 49.08 | 17.8 | 25.1 | -21.9 | 0.619 |
| 22 | Superior temporal | 0.037 | 45.93 | 47 | -29.9 | 7.7 | 0.626 |
| 23 | Insula | 0.038 | 38.27 | 39 | -0.5 | -17.6 | 0.653 |
| 24 | Superior temporal | 0.041 | 37.37 | 45.4 | -22.9 | -9.6 | 0.653 |
| 25 | Superior parietal | 0.039 | 36.46 | 24.3 | -40.6 | 53.8 | 0.655 |
| 26 | Superior frontal | 0.038 | 34.89 | 13.8 | -6.5 | 67.4 | 0.658 |
| 27 | Superior frontal | 0.035 | 32.4 | 21.9 | -2.1 | 53.3 | 0.664 |
| 28 | Inferior parietal | 0.046 | 29.07 | 42.1 | -78.6 | 18.4 | 0.677 |
| 29 | Precentral | 0.043 | 23.92 | 15.7 | -17.6 | 67.2 | 0.69 |
| 30 | Lateral occipital | 0.040 | 20.41 | 42.3 | -62.9 | 2.4 | 0.702 |
| 31 | Posterior cingulate | 0.037 | 15.84 | 10.1 | -6.4 | 41.9 | 0.727 |
| 32 | Bankssts | 0.045 | 6.61 | 46.5 | -36.6 | 5.4 | 0.762 |
| 33 | Paracentral | 0.048 | 4.39 | 12 | -39.1 | 62.4 | 0.771 |
| 34 | Inferior parietal | 0.050 | 1.6 | 40.2 | -52 | 40.2 | 0.786 |
| 35 | Posterior cingulate | 0.050 | 0.38 | 9.5 | -29.2 | 39 | 0.791 |
|  |  |  |  |  |  |  |  |

All cluster contrasts were Cognitively Normal > Alzheimer's disease. ROI = Region of Interest, Area = area of the cluster in mm2, [MNI_X MNI_Y MNI_Z] = MNI305 coordinates of the cluster, *P* Max = maximum *P* value within the cluster prior to correction for multiple comparisons, CW *P* = cluster-wise *P* value corrected for multiple comparisons using permutation, Bankssts = banks of the superior temporal sulcus

| **Supplemental Table 3.** **mGluR5 binding in all FreeSurfer regions** | | | | | | | | | |
| --- | --- | --- | --- | --- | --- | --- | --- | --- | --- |
|  | ***BP*_ND_ – left hemisphere** | |  | | ***BP*_ND_ – right hemisphere** | | | |  |
| **Region** | **CN (n = 15)**  **Mean** **(SD)** | **AD (n = 16)**  **Mean** **(SD)** | | ***P*** | | **CN (n = 15)**  **Mean** **(SD)** | **AD (n = 16)**  **Mean** **(SD)** | ***P*** | |
| Frontal pole | 2.65 (0.84) | 2.34 (0.23) | | 0.20 | | 2.66 (0.9) | 2.35 (0.31) | 0.26 | |
| Superior frontal gyrus | 2.44 (0.77) | 2.16 (0.27) | | 0.27 | | 2.38 (0.76) | 2.12 (0.26) | 0.28 | |
| Rostral middle frontal gyrus | 2.74 (0.8) | 2.49 (0.32) | | 0.34 | | 2.65 (0.9) | 2.35 (0.38) | 0.30 | |
| Caudal middle frontal gyrus | 2.28 (0.75) | 1.98 (0.3) | | 0.22 | | 2.19 (0.64) | 1.9 (0.35) | 0.18 | |
| Pars orbitals | 2.62 (0.95) | 2.35 (0.32) | | 0.35 | | 2.44 (0.89) | 2.21 (0.27) | 0.39 | |
| Pars opercularis | 2.66 (0.85) | 2.38 (0.28) | | 0.27 | | 2.48 (0.82) | 2.22 (0.32) | 0.33 | |
| Pars triangularis | 2.58 (0.88) | 2.35 (0.33) | | 0.42 | | 2.56 (0.91) | 2.23 (0.34) | 0.23 | |
| Lateral orbitofrontal | 2.45 (0.84) | 2.24 (0.28) | | 0.42 | | 2.34 (0.78) | 2.06 (0.33) | 0.25 | |
| Medal orbitofrontal | 2.74 (0.97) | 2.46 (0.35) | | 0.35 | | 2.63 (0.86) | 2.42 (0.37) | 0.45 | |
| Temporal pole | 2.27 (0.8) | 1.97 (0.34) | | 0.27 | | 2.2 (0.73) | 1.92 (0.47) | 0.28 | |
| Entorhinal cortex | 1.89 (0.7) | 1.48 (0.3) | | 0.06 | | 1.8 (0.69) | 1.24 (0.43) | 0.02* | |
| Parahippocampal cortex | 2.03 (0.69) | 1.61 (0.3) | | 0.05 | | 1.93 (0.56) | 1.53 (0.4) | 0.04* | |
| Hippocampus | 1.89 (0.61) | 1.49 (0.3) | | 0.04* | | 1.75 (0.55) | 1.42 (0.37) | 0.09 | |
| Amygdala | 2.12 (0.77) | 1.8 (0.4) | | 0.24 | | 2.03 (0.67) | 1.65 (0.46) | 0.10 | |
| Inferior temporal gyrus | 2.6 (0.77) | 2.2 (0.33) | | 0.09 | | 2.45 (0.76) | 2.04 (0.44) | 0.11 | |
| Fusiform gyrus | 2.47 (0.73) | 2.06 (0.31) | | 0.07 | | 2.34 (0.7) | 1.97 (0.39) | 0.10 | |
| Middle temporal gyrus | 2.72 (0.88) | 2.31 (0.35) | | 0.12 | | 2.49 (0.81) | 2.13 (0.42) | 0.17 | |
| Banks of the superior temporal sulcus | 2.69 (0.82) | 2.17 (0.33) | | 0.04* | | 2.38 (0.84) | 1.95 (0.34) | 0.10 | |
| Superior temporal gyrus | 2.46 (0.81) | 2.14 (0.28) | | 0.18 | | 2.28 (0.8) | 2.01 (0.39) | 0.29 | |
| Transverse temporal gyrus | 2.33 (0.8) | 2.03 (0.28) | | 0.20 | | 2.08 (0.75) | 1.79 (0.36) | 0.24 | |
| Supramarginal gyrus | 2.57 (0.81) | 2.24 (0.32) | | 0.18 | | 2.38 (0.72) | 2.1 (0.35) | 0.26 | |
| Insular cortex | 2.36 (0.75) | 2.07 (0.3) | | 0.21 | | 2.22 (0.7) | 1.93 (0.37) | 0.22 | |
| Rostral anterior cingulate cortex | 2.69 (0.85) | 2.66 (0.47) | | 0.91 | | 2.55 (0.76) | 2.5 (0.38) | 0.96 | |
| Caudal anterior cingulate cortex | 2.4 (0.64) | 2.32 (0.4) | | 0.89 | | 2.49 (0.76) | 2.25 (0.35) | 0.36 | |
| Posterior cingulate cortex | 2.41 (0.75) | 2.15 (0.26) | | 0.28 | | 2.49 (0.76) | 2.19 (0.29) | 0.22 | |
| Isthmus of the cingulum | 1.86 (0.71) | 1.54 (0.24) | | 0.13 | | 1.93 (0.65) | 1.56 (0.26) | 0.05 | |
| Precuneus | 2.24 (0.61) | 2 (0.29) | | 0.21 | | 2.24 (0.64) | 1.99 (0.28) | 0.20 | |
| Paracentrual gyrus | 1.67 (0.57) | 1.46 (0.23) | | 0.26 | | 1.67 (0.5) | 1.46 (0.27) | 0.22 | |
| Postcentral gyrus | 1.99 (0.64) | 1.8 (0.23) | | 0.35 | | 1.9 (0.61) | 1.72 (0.27) | 0.38 | |
| Precentral gyrus | 1.89 (0.67) | 1.66 (0.24) | | 0.27 | | 1.8 (0.6) | 1.6 (0.27) | 0.31 | |
| Superior parietal lobule | 2.07 (0.61) | 1.76 (0.26) | | 0.10 | | 1.96 (0.49) | 1.73 (0.28) | 0.17 | |
| Inferior parietal lobule | 2.45 (0.68) | 2.15 (0.26) | | 0.14 | | 2.39 (0.68) | 2.06 (0.39) | 0.16 | |
| Lateral occipital cortex | 2.06 (0.64) | 1.82 (0.29) | | 0.21 | | 2.02 (0.55) | 1.79 (0.28) | 0.20 | |
| Cuneus | 1.92 (0.59) | 1.76 (0.23) | | 0.34 | | 1.88 (0.52) | 1.73 (0.25) | 0.39 | |
| Pericalcarine cortex | 1.82 (0.61) | 1.78 (0.27) | | 0.84 | | 1.87 (0.59) | 1.76 (0.21) | 0.54 | |
| Lingual gyrus | 1.96 (0.7) | 1.72 (0.21) | | 0.23 | | 1.81 (0.53) | 1.6 (0.24) | 0.18 | |
| Thalamus | 1.02 (0.51) | 0.83 (0.17) | | 0.23 | | 1.04 (0.51) | 0.84 (0.17) | 0.20 | |
| Cuadate | 1.96 (0.77) | 1.86 (0.39) | | 0.72 | | 2.02 (0.85) | 1.76 (0.39) | 0.34 | |
| Putamen | 2.55 (0.87) | 2.32 (0.32) | | 0.36 | | 2.45 (0.83) | 2.19 (0.3) | 0.29 | |
| Pallidum | 0.71 (0.42) | 0.66 (0.16) | | 0.73 | | 0.76 (0.45) | 0.59 (0.14) | 0.16 | |
| Nucleus accumbens | 2.89 (0.97) | 2.82 (0.41) | | 0.89 | | 2.95 (1.03) | 2.78 (0.42) | 0.67 | |
| Ventral diencephalon | 0.17 (0.24) | 0.09 (0.11) | | 0.31 | | 0.19 (0.28) | 0.1 (0.11) | 0.27 | |

Data are mean (SD). *P*-values are for post hoc two-tailed, unpaired *t*-tests (uncorrected for multiplicity) between CN and AD diagnostic groups. **P* < 0.05 Abbreviations: *BP*_ND_, binding potential of [^18^F]FPEB in regions of interest calculated with a cerebellum reference region; CN, cognitively normal; AD, Alzheimer’s disease.

**
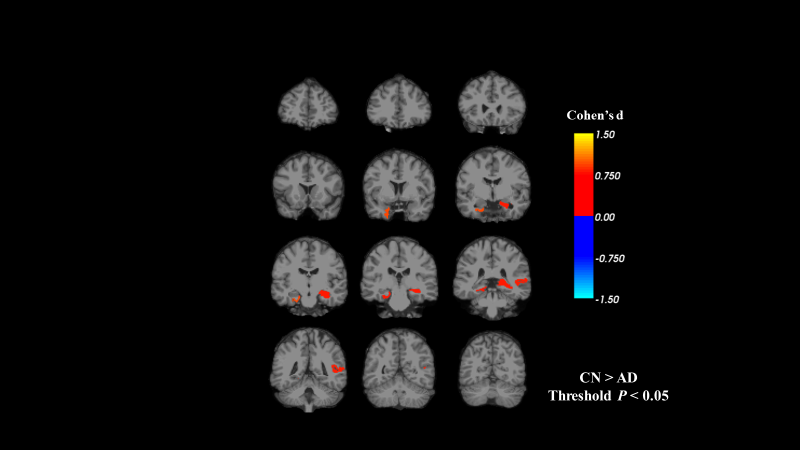
**

**Supplemental Figure 1.** **Effect size maps of [^18^F]FPEB binding (*BP*_ND_) to mGluR5 between AD and CN groups.** Effect sizes for differences in AD compared to CN groups were calculated for all FreeSurfer regions. The color scale represents Cohen’s d for the comparison between AD and CN groups. Effect size statistics are displayed only for regions that had an uncorrected *P* < 0.05. Contrast is for CN>AD. Abbreviations: CN, cognitively normal; AD, Alzheimer’s disease; PET, positron emission tomography; *BP*_ND_, binding potential.
